# Characterization of *ex vivo* and *in vivo* intraoperative neurosurgical confocal laser endomicroscopy imaging

**DOI:** 10.3389/fonc.2022.979748

**Published:** 2022-08-24

**Authors:** Yuan Xu, Irakliy Abramov, Evgenii Belykh, Giancarlo Mignucci-Jiménez, Marian T. Park, Jennifer M. Eschbacher, Mark C. Preul

**Affiliations:** ^1^ The Loyal and Edith Davis Neurosurgical Research Laboratory, Department of Neurosurgery, Barrow Neurological Institute, St. Joseph’s Hospital and Medical Center, Phoenix, AZ, United States; ^2^ Department of Neuropathology, Barrow Neurological Institute, St. Joseph’s Hospital and Medical Center, Phoenix, AZ, United States

**Keywords:** brain tumor, confocal laser endomicroscopy, fluorescein sodium, fluorescence, glioma, intraoperative imaging, meningioma

## Abstract

**Background:**

The new US Food and Drug Administration-cleared fluorescein sodium (FNa)-based confocal laser endomicroscopy (CLE) imaging system allows for intraoperative on-the-fly cellular level imaging. Two feasibility studies have been completed with intraoperative use of this CLE system in *ex vivo* and *in vivo* modalities. This study quantitatively compares the image quality and diagnostic performance of *ex vivo* and *in vivo* CLE imaging.

**Methods:**

Images acquired from two prospective CLE clinical studies, one *ex vivo* and one *in vivo*, were analyzed quantitatively. Two image quality parameters – brightness and contrast – were measured using Fiji software and compared between *ex vivo* and *in vivo* images for imaging timing from FNa dose and in glioma, meningioma, and intracranial metastatic tumor cases. The diagnostic performance of the two studies was compared.

**Results:**

Overall, the *in vivo* images have higher brightness and contrast than the *ex vivo* images (p < 0.001). A weak negative correlation exists between image quality and timing of imaging after FNa dose for the *ex vivo* images, but not the *in vivo* images. *In vivo* images have higher image quality than *ex vivo* images (p < 0.001) in glioma, meningioma, and intracranial metastatic tumor cases. *In vivo* imaging yielded higher sensitivity and negative predictive value than *ex vivo* imaging.

**Conclusions:**

In our setting, *in vivo* CLE optical biopsy outperforms *ex vivo* CLE by producing higher quality images and less image deterioration, leading to better diagnostic performance. These results support the *in vivo* modality as the modality of choice for intraoperative CLE imaging.

## Introduction

Intraoperative diagnosis of brain tumors primarily relies on frozen section pathology, which is the histological interpretation of hematoxylin and eosin (H&E)-stained frozen section preparation of biopsied tissue under light microscopy. However, the heterogeneous nature of certain brain tumors and the limited number of biopsy specimens make it prone to sampling errors. In addition, the freezing process could induce freezing artifact or distort cell morphology, rendering it challenging to interpret the pathology precisely (1). Unless histology and pathology processing and interpretation are immediately adjacent to the operating room, the complete process of tissue transfer, preparation, and inspection often takes 20-40 minutes or longer (2).

Confocal laser endomicroscopy (CLE), using a new Food and Drug Administration (FDA)-cleared (3) fluorescein sodium (FNa)-based imaging system, provides the possibility of intraoperative real-time, on-the-fly cellular level imaging. Combined with the application of surgical fluorescence, CLE adds the option of an optical biopsy of tissue histology into neurosurgeons’ toolbox (4–6). In the past decade, a probe-based CLE system has been extensively studied in animal models with different pathologies, producing promising results (7–11) before human subject studies (10, 12–16). It has been shown that this technology has the potential to assume a substantial role in intraoperative diagnosis for brain tumor surgery.

For the past 2 years, two prospective human subject feasibility studies, one *ex vivo* (17) and one *in vivo* (18), using intraoperative CLE imaging in a neurosurgical setting have been completed in our center. Analysis of FNa dosing has been based on the results of the *ex vivo* study (17, 19), which is only now able to be contrasted with results of imaging from the *in vivo* study. The original design and intent of the CLE system for neurosurgery were for *in vivo* use. Thus, assessment of the recently acquired *in vivo* study images side by side with the previous *ex vivo* images is crucial for full appreciation of the feasibility of the CLE system. This retrospective image quality analysis aims to quantitatively compare the image quality and diagnostic performance of the two CLE imaging modalities to understand the optimal scenario for CLE application.

## Materials and methods

### Study design

Two prospective studies were conducted at Barrow Neurological Institute, St. Joseph’s Hospital and Medical Center (Phoenix, Arizona, USA). Between August 2016 and May 2019, samples from 43 adult patients (≥ 18 years) undergoing FNa fluorescence-guided brain tumor surgery were imaged *ex vivo* with CLE (*ex vivo* feasibility study) (17). Between May 2020 and July 2021, 30 adult patients (≥18 years) who had brain tumor surgery were imaged *in vivo* with CLE after FNa administration during the operation (18, 20). Both studies were approved by the St. Joseph’s Hospital and Medical Center Institutional Review Board for Human Research (IRB No. 10BNI130 and PHX-19-500-403-80-12, respectively). Informed consent was obtained from each patient. Exclusion criteria included (1) prior history of FNa hypersensitivity, (2) renal failure, (3) age < 18 years, (4) pregnancy or breastfeeding, and (5) inability to provide informed consent.

For both studies, standard intraoperative techniques, including intraoperative imaging-based neuronavigation, surgical microscope, endoscopic assistance, and intraoperative brain mapping, were used as needed. No intraoperative clinical decision was made based on the CLE images.

### Fluorescein sodium administration

For the *ex vivo* study, FNa was administered intravenously at the induction of anesthesia at a dose of 2 mg/kg for glioma and meningioma cases and 5 mg/kg for metastatic tumor cases. An additional 5-mg/kg dose was given if the neurosurgeon considered it necessary. One patient received a one-time dose of 40 mg/kg of FNa at the induction of anesthesia (17). For the *in vivo* study, a 5-mg/kg dose of FNa was given intravenously approximately 5 minutes before the first images were acquired, and surgeons did not order to redose FNa (18, 20).

### Image acquisition

Optical biopsies were acquired with a CLE imaging system (CONVIVO, Carl Zeiss Meditec AG, Jena, Germany) using a 488 nm excitation laser. As described by Belykh et al., when used with a green bandpass filter with laser power set at 50%, the gain set at 2400, it produces the highest quality images with 1920 × 1080-pixel resolution at 1.26 sec per frame acquisition speed (21). For the *ex vivo* study, CLE images were acquired from fresh tissue samples removed as a standard neurosurgical procedure using a probe affixed by a probe holder in an upright position. A 517.5 to 572.5-nm bandpass filter at 1× zoom with automatic gain was used, producing images with 1920 × 1080-pixel resolution and a 267 × 475-μm field of view (17). For the *in vivo* study, CLE images were acquired *in situ* during the operation using a handheld probe coregistered with the intraoperative navigation system. A 515 to 577-nm bandpass filter was used, with the gain maintained at 2400. The resulting images were 1920 × 1080-pixel resolution, with a 267 × 475-μm field of view (18, 20).

### Image processing and analysis

As previously described (19), all images collected from the two above-mentioned studies were processed and analyzed with Fiji software (22) (https://imagej.net/software/fiji/). For each image, two parameters (brightness and contrast) were assessed to determine image quality. Brightness was defined as the mean gray value of all the pixels of the image. Contrast was defined as the standard deviation (SD) of the pixel gray values. Images with total blackout signals were sampled from both studies. Their mean brightness and contrast values and SDs were calculated, and the mean + 2SD was used as the lower threshold for exclusion from all analyses. The quality of all images was compared between images from the two studies. A separate comparison for brightness and contrast was performed between images from both studies acquired 120 minutes or more after the last FNa dose. The image quality of images from the *ex vivo* study after an FNa redose was compared to that of the *in vivo* images. The images from *in vivo* and *ex vivo* glioma and meningioma cases were compared for brightness and contrast.

### Image interpretation

The images from both studies were interpreted by three CLE-experienced neurosurgeons and a CLE-experienced neuropathologist. Three interpretation categories were used: lesional, nonlesional, and nondiagnostic. Lesional images were defined as CLE images with features similar to or representative of a mass lesion. Nonlesional images were defined as CLE images that did not contain identifiable features of a mass lesion. Nondiagnostic images were defined as CLE images lacking identifiable features necessary for interpretation. Pathology diagnoses based on permanent H&E-stained sections were used as the gold standard for calculating sensitivity, specificity, positive predictive value, and negative predictive value.

### Statistical analysis

Statistical analysis was performed using GraphPad Prism 9.3 (GraphPad Software Inc., La Jolla, CA). Continuous variables were presented as means with SDs. Categorical variables were presented as counts and percentages. The differences in brightness and contrast between images from the two studies were compared using the Mann-Whitney U test. The correlation between image quality and time after the last FNa dose was characterized by the Spearman correlation. A p-value < 0.05 was considered statistically significant.

## Results

### Descriptive analysis

For the *ex vivo* study, 43 patients with 118 optical biopsies were included in the analysis. Of 17,951 available images, 14,638 were analyzed, with 3,313 unusable images excluded from the analysis due to signal blackout or significant artifacts. For the *in vivo* study, 30 patients with 87 optical biopsies were included. Of 10,125 available images, 6,975 images were analyzed, with 3,151 unusable images excluded. Images with blackout signals were sampled from both studies, and their brightness and contrast values were analyzed (range: 7.56-25.22 and 4.10-13.78, respectively; median: 13.12 and 6.48, respectively). Mean (SD) brightness and contrast values were calculated to be 14.61 (5.85) and 7.38 (2.93), respectively. A mean + 2SD upper threshold (brightness < 26.30, contrast < 13.24) was used to exclude the images with blackout signals. All optical biopsies were confirmed by permanent section pathology analyses (**Table 1**). Six patients from the *ex vivo* study received an additional FNa dose at some point during the surgery. Data on the diagnostic performance of *in vivo* and *ex vivo* CLE imaging were published previously (17, 20) and are presented in **Table 2**.

**Table 1:** An overview of pathology types of the cases from the *in vivo* and *ex vivo* studies.

Study	Glioma, n	Meningioma, n	Other pathologies, n
*In vivo* study	13	5	Choroid plexus papilloma, 1
			Hemangioblastoma, 1
			Intracranial metastatic tumor, 3
			Mature teratoma, 1
			Perineuroma, 1
			Pineocytoma, 1
			Treatment effect, 4
			Vestibular schwannoma, 1
*Ex vivo* study	29	7	Arteriovenous malformation, 1
			Choroid plexus carcinoma, 1
			Craniopharyngioma, 1
			Intracranial metastatic tumor, 3
			Vestibular schwannoma, 1

**Table 2:** Diagnostic performance of confocal laser endomicroscopy (CLE) in all cases and glioma cases.

Variable	*Ex vivo* study		*In vivo* study	
	All Samples	Glioma	All Samples	Glioma
Sensitivity	72 (62-80)	66 (55-76)	90 (78-96)	91 (72-98)
Specificity	90 (67-98)	94 (69-100)	94 (74-100)	100 (65-100)
Positive predictive value	97 (90-100)	98 (89-100)	97 (87-100)	100 (84-100)
Negative predictive value	38 (25-54)	37 (23-53)	81 (60-92)	78 (45-96)

All data are presented as percent and 95% confidence intervals.

Permanent hematoxylin and eosin–stained section pathology was used as the gold standard. All CLE digital biopsies were interpreted by a single experienced neuropathologist.

### Image quality analysis

#### Overall comparison

**Figure 1:**
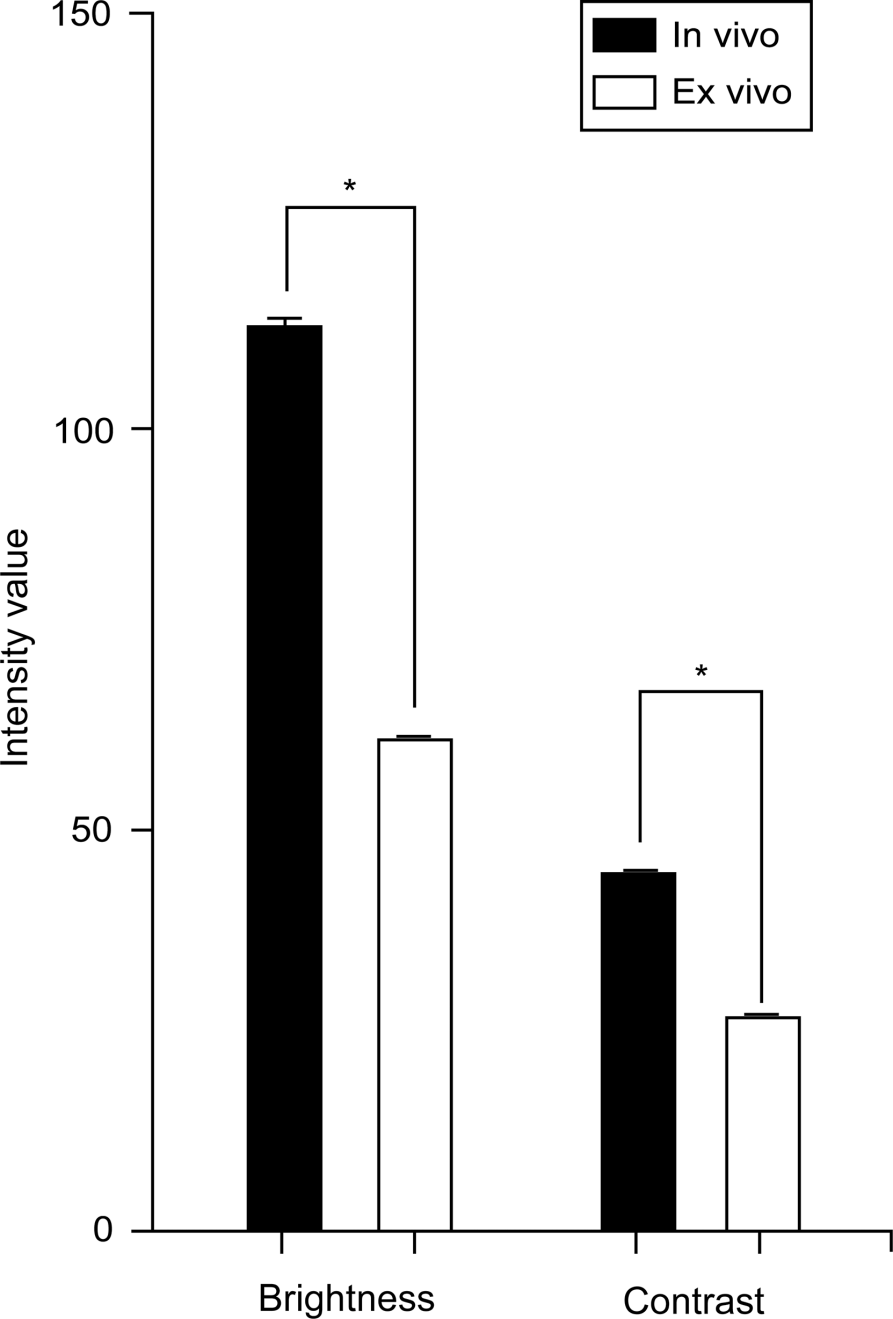
Bar chart showing a comparison of overall mean (SD) brightness and contrast of *in vivo* and *ex vivo* images. *In vivo* images had a mean brightness of 112.1 (26.9) and contrast of 44.7 (8.0). *Ex vivo* images had a mean brightness of 60.7 (23.6) and a contrast of 26.8 (7.9). *In vivo* images have higher brightness and contrast than *ex vivo* images (p < 0.001). Values are reported in optical density units defined by the Fiji software. Asterisk indicates p < 0.001. *Used with permission from Barrow Neurological Institute, Phoenix, Arizona*.

**Figure 2:**
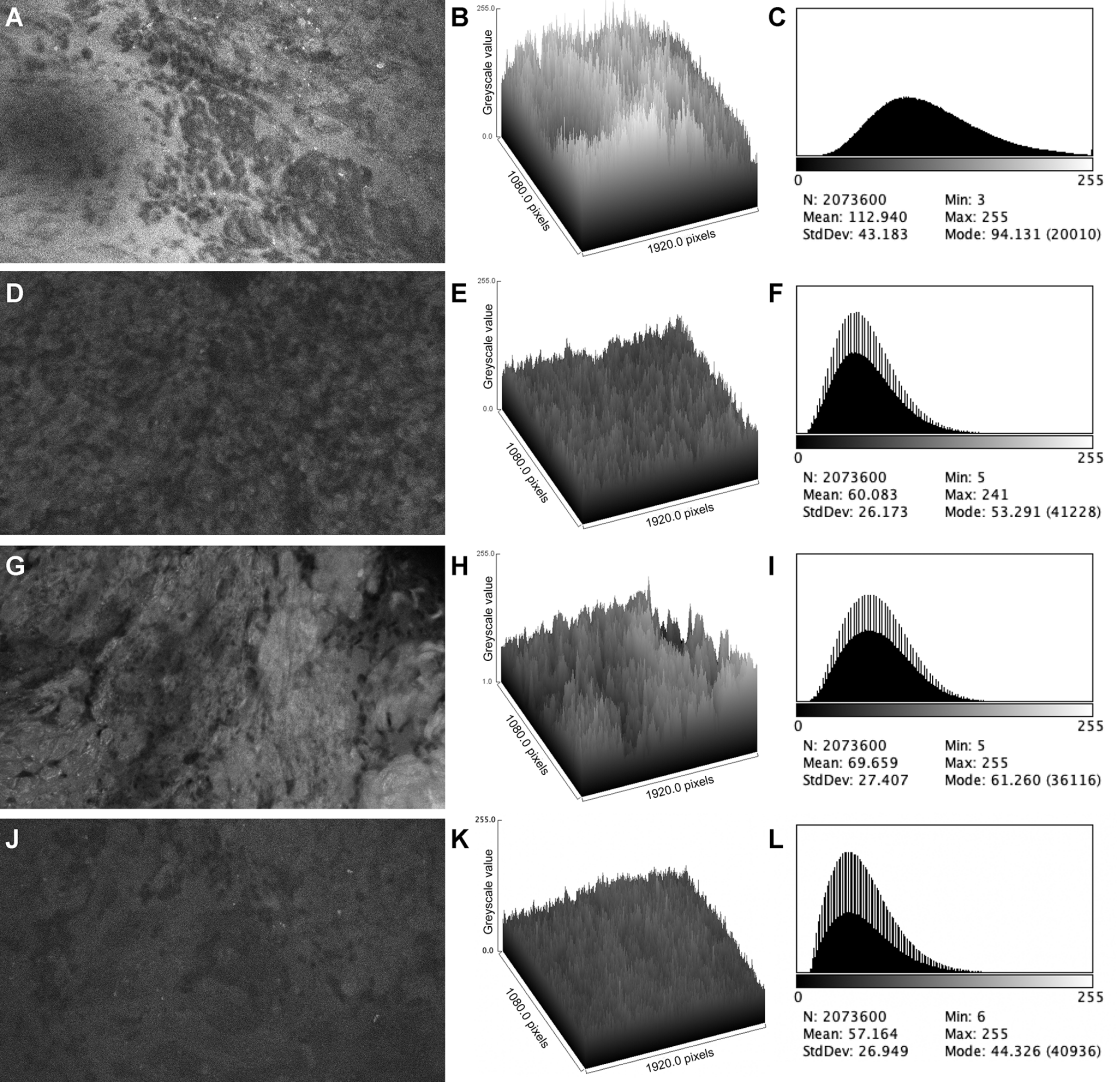
Examples of confocal laser endomicroscopy images and data from *in vivo* and *ex vivo* studies. *In vivo* image and data **(A–C)** show a brighter background and more perceivable cellular structure than the corresponding *ex vivo* image and data **(D–F)**. *Ex vivo* image and data from a patient who had an additional FNa dose **(G–I)** shows improved overall brightness and contrast compared to the image from the same patient before FNa redosing **(J–L)**. Mean, brightness; StdDev, brightness; Min and Max, minimum and maximum gray value of pixels in the image; Mode, most frequent occurring gray value in the image. Values are reported in optical density units defined by Fiji software. *Used with permission from Barrow Neurological Institute, Phoenix, Arizona*.

**Figure 3:**
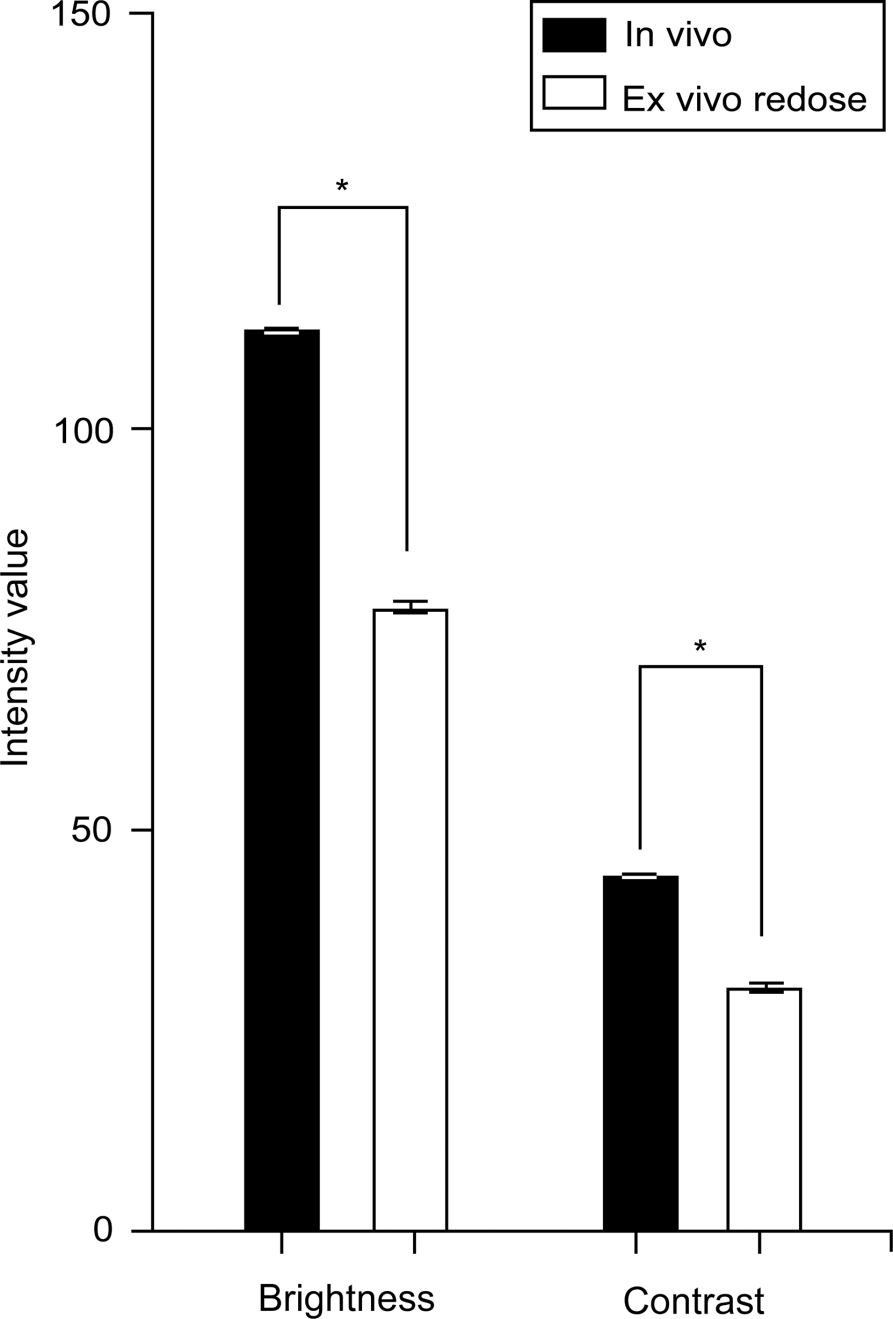
Bar plot showing the brightness and contrast of *ex vivo* images from patients that were redosed with fluorescein sodium. The mean brightness (77.8 [19.9]) and contrast (30.9 [7.5]) are inferior to those from the *in vivo* study. Values are reported in optical density units defined by Fiji software. Asterisk indicates p < 0.001. *Used with permission from Barrow Neurological Institute, Phoenix, Arizona*.

The images from the *in vivo* study had significantly higher brightness and contrast values than the images from the *ex vivo* study (brightness: 112.1 [26.9] vs. 60.7 [23.6], p < 0.001; contrast: 44.7 [8.0] vs. 26.8 [7.9], p < 0.001, **Figures 1,  2A–F**). The mean (SD) brightness and contrast of images from the *ex vivo* study with FNa redosing (**Figures 2G–L**) were 77.8 (19.9) and 30.9 (7.5), lower than those of the images from the *in vivo* study (p < 0.001, **Figure 3**).

#### Timing of imaging

**Figure 4:**
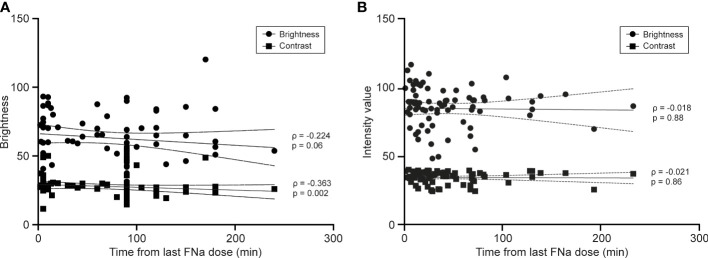
Line graphs showing the correlation between image quality and image timing. **(A)** For the *ex vivo* study, there is a small negative correlation between image contrast and the time interval between fluorescein sodium dosing and imaging (ρ = -0.363, p = 0.002). The result for image brightness and timing of imaging was not statistically significant (ρ = -0.224, p = 0.06). **(B)** For the *in vivo* study, no correlation was observed between image brightness (ρ = -0.018, p = 0.88) and contrast (ρ = -0.021, p = 0.86) and timing of imaging. *Used with permission from Barrow Neurological Institute, Phoenix, Arizona*.

**Figure 5:**
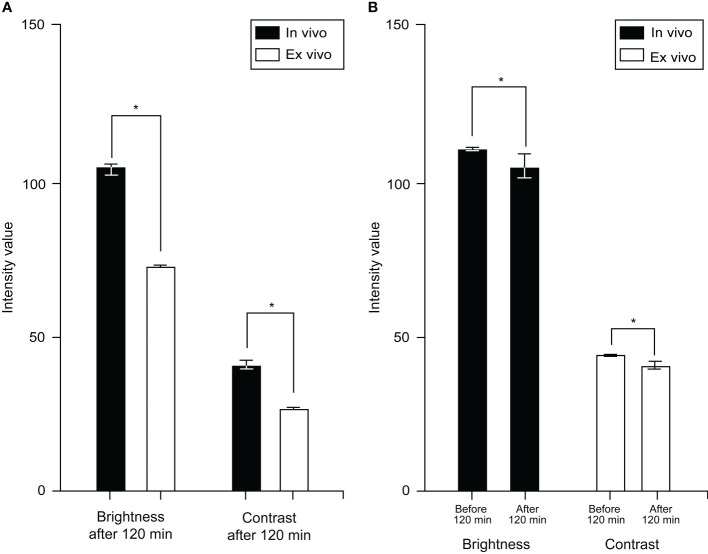
Bar chart showing mean (SD) brightness and contrast of images acquired at different time intervals after fluorescein sodium (FNa) dosing. **(A)** Comparison of images acquired later than 120 min after FNa dosing in both studies; the mean brightness (*in vivo* vs. *ex vivo*: 105.1 [18.9] vs. 72.4 [24.9], p < 0.001) and contrast (*in vivo* vs. *ex vivo*: 41.7 [8.7] vs. 27.1 [8.5], p < 0.001) of the *in vivo* images are significantly higher than those of the *ex vivo* images. **(B)** Compared to the images acquired earlier than 120 minutes after FNa dose, the images acquired later than 120 minutes had a slight decrease in brightness (112.2 [27.0] to 105.1 [18.9], p < 0.001) and contrast (44.74 [8.0] to 41.07 [8.6], p < 0.001). Values are reported in optical density units defined by Fiji software. Asterisk indicates p < 0.001. *Used with permission from Barrow Neurological Institute, Phoenix, Arizona*.

The mean (SD) time interval between image acquisition and the last FNa dose for patients in the *ex vivo* study was significantly longer than that of the *in vivo* study (73.7 [55.8] min vs. 37.2 [37.8] min, p = 0.001). For the *ex vivo* study, a weak negative correlation was found between image contrast and time from the last FNa dose (ρ = -0.36, p = 0.002, **Figure 4A**). Similarly, though not statistically significant, a negative trend was found between image brightness and timing of image acquisition (ρ = -0.22, p = 0.06, **Figure 4A**). For the *in vivo* study, no correlation was identified between either image brightness or contrast and time from last FNa dose (brightness: ρ = -0.02, p = 0.88; contrast: ρ = -0.02, p = 0.86, **Figure 4B**). When comparing all images acquired 120 minutes after the last FNa dose, images from the *in vivo* study have significantly higher brightness and contrast than images from the *ex vivo* study (brightness: 105.1 [18.9] vs. 72.4 [24.93], p < 0.001, contrast: 41.7 [8.7] vs. 27.1 [8.5], p < 0.001, **Figure 5**).

#### Images from glioma, meningioma, and intracranial metastatic tumor cases

**Figure 6:**
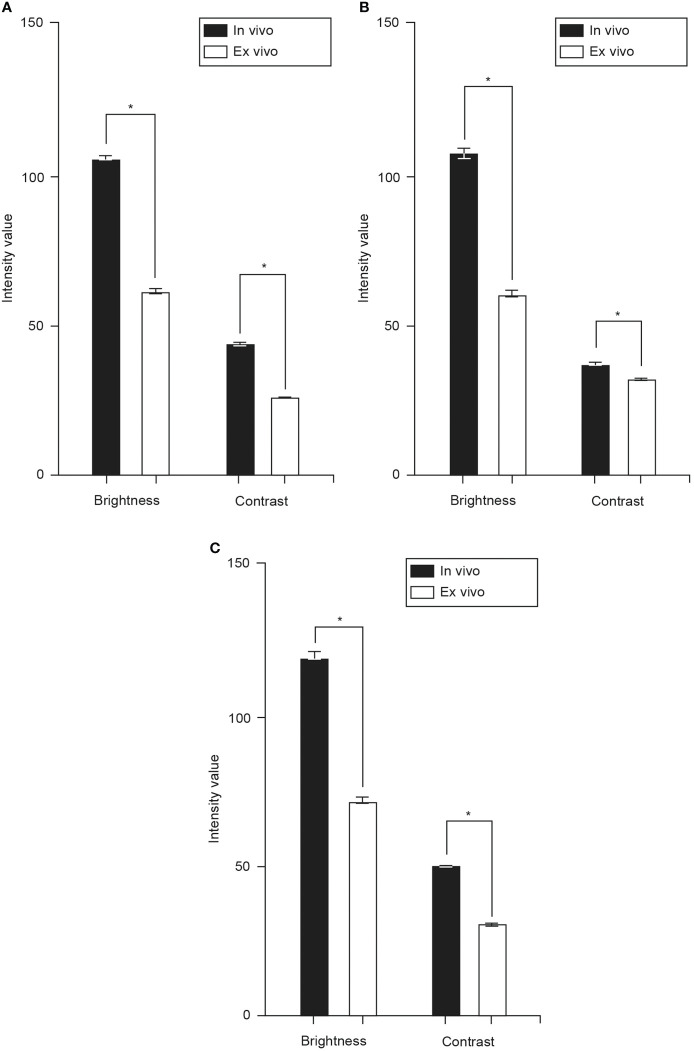
Bar charts showing mean brightness and contrast of images from glioma **(A)**, meningioma **(B)**, and intracranial metastatic tumor **(C)** cases from both studies. The mean brightness and contrast of the *in vivo* images from glioma (brightness: 105.2 [25.8] vs. 60.6 [8.1], p < 0.001; contrast: 43.2 [24.9] vs. 26.5 [6.9], p < 0.001), meningioma (brightness: 106.3 [26.0] vs. 60.0 [18.8], p < 0.001; contrast: 36.7 [8.0] vs. 31.7 [8.9], p < 0.001), and intracranial metastatic tumor (brightness: 118.2 [26.5] vs. 70.6 [25.1], p < 0.001; contrast: 49.6 [4.9] vs. 30.1 [6.8], p < 0.001, Figure 6C values are significantly higher than those of the *ex vivo* images. Values are reported in optical density units defined by Fiji software. Asterisk indicates p < 0.001. *Used with permission from Barrow Neurological Institute, Phoenix, Arizona*.

Brightness and contrast values of the images from glioma cases were significantly higher in the *in vivo* study compared to the *ex vivo* study (brightness: 105.2 [25.8] vs. 60.6 [8.1], p < 0.001; contrast: 43.2 [24.9] vs. 26.5 [6.9], p < 0.001, **Figure 6A**). The same difference was found in images from meningioma cases (brightness: 106.3 [26.0] vs. 60.0 [18.8], p < 0.001; contrast: 36.7 [8.0] vs. 31.7 [8.9], p < 0.001, **Figure 6B**) and intracranial metastatic tumor cases (brightness: 118.2 [26.5] vs. 70.6 [25.1], p < 0.001; contrast: 49.6 [4.9] vs. 30.1 [6.8], p < 0.001, **Figure 6C**).

## Discussion

Both *ex vivo* and *in vivo* modalities produce intraoperative images of tissue histoarchitecture, providing valuable diagnostic clues. This retrospective study is the first to directly compare the two modalities by their image quality and diagnostic performance.

The CLE system used in this study was initially developed as a diagnostic device for gastrointestinal endoscopies (23). Investigation for CLE application with the system ultimately designed and approved for clinical neurosurgery started in 2008 (24). The FDA-cleared CLE system used for these studies consists of a handheld probe and a workstation with a display monitor. The term confocal refers to the alignment of the light source and the collection system in the same specimen plane. After the laser illuminates the specimen, the tissue emits fluorescence, which is detected by the collection system for image generation (25). The current generation CLE device we used was designed specifically to be integrated into the neurosurgical workflow for intraoperative use. Compared to the previous generation CLE system, it has a sterile sheath, a probe (approximately the size of a neurosurgical suction tip) optimally designed to be held by a neurosurgeon, and an improved image display (21).

In order to achieve optimal tissue fluorescence, certain fluorescent contrasts are required. FNa, 5-aminolevulinic acid (5-ALA), and indocyanine green (ICG) have been used *in vivo* in neurosurgery, although not all of them are approved in all countries. FNa and 5-ALA have been extensively studied for their use in brain tumor surgery, mostly with wide-field optical imaging (i.e., using fluorescence detection modules adapted to a neurosurgical operating microscope). They require a disrupted blood-brain barrier for extravasation to reach the tumor tissue, so they are less effective for low-grade gliomas (26). 5-ALA is usually administered orally at least 3 hours before the surgery since it needs to be converted to its fluorescent metabolite, protoporphyrin IX (15). On the contrary, FNa can be administered intravenously merely a few minutes before imaging because of its rapid distribution (27). ICG has seen wide use in cerebrovascular surgery for ICG angiography. So far, ICG fails to provide sufficient fluorescence for tumor visualization with standard CLE equipment and has not been investigated in humans to the same extent as wide-field imaging.

Besides employing FNa, CLE systems have been developed using 5-ALA and ICG, although these are not clinically approved (7, 28). Only FNa is compatible with the FDA-cleared CLE system used in these *ex vivo* and *in vivo* studies. The main system construction limitation is that FNa, 5-ALA, and ICG require illumination systems of different wavelengths: FNa has an excitation wavelength in the blue range of 475-490 nm, and the excitation wavelength of 410 nm of 5-ALA is close to that of FNa, and ICG functions at an excitation of 750-800 nm within the near-infrared light range. There are no clinical systems that support a wide range of excitation wavelengths.

### Imaging modality: *In vivo* vs. *ex vivo*

With *ex vivo* CLE imaging, a piece of tissue must be resected before being imaged. This tissue extraction process is identical to that of a regular biopsy. Potentially after that, CLE produces interpretable cellular-level microscopic images within seconds, significantly faster than standard frozen section pathology. In contrast, with *in vivo* CLE imaging, the probe is placed and moved directly in contact with the tissue in situ. This unique advantage enables the neurosurgeon to visualize in real-time on-the-fly and even scan the histoarchitecture of the area of interest before any tissue is extracted. Such a technique may increase the positive yield and/or limit the need for frozen section biopsy. This could be especially useful in cases of eloquent region lesions, where a preliminary pathology diagnosis may minimize unnecessary tissue injury and thus reduce the risk of neurological deficit. In cases of uncertain preoperative diagnosis, a nonneoplastic diagnosis made by CLE could negate the need for surgical resection.

Although the choice of the fluorophore used for *in vivo* CLE imaging is restricted to some extent due to systemic toxicity, a more comprehensive range of fluorophores can be used for *ex vivo* imaging. Although only FNa was used in the *ex vivo* clinical feasibility study with the FDA-cleared CLE system, acriflavine, acridine orange, and cresyl violet are fluorophores in the blue to yellow light range that have been investigated for *ex vivo* CLE imaging using a system that was a progenitor to the current clinically-approved system (29, 30). They have the unique advantage of topical administration that rapidly stains extracellular, cellular membrane, and intracellular structures, including nuclear, making them suitable for *ex vivo* CLE tissue analysis in a side bench fashion. Due to their toxicity profile, they are not approved for *in vivo* use. These fluorophores can be imaged with the blue light CLE system used in an *ex vivo* mode, but they cannot be administered to the patient for *in vivo* CLE imaging because of their imputed mutagenic characteristics.

Although the main design purpose of the CLE system is for real-time *in vivo* functionality, the combination of the *ex vivo* imaging and aforementioned fluorophores, or other visualizable specific molecular labeling not of *in vivo* compatibility, can potentially advocate the use of a CLE system as a backbench high-resolution cellular microscope of the resected tissue within seconds. Although benchtop confocal microscope systems that have several lasers may provide a more comprehensive tissue analysis, *ex vivo* CLE might provide a timely advantage for augmenting frozen sections, especially where CLE systems may support blue and near-infrared light imaging (31, 32). Care must be taken, however, to ensure there is no contamination into the operative field or patient tissue exposure with these fluorophores.

### Image acquisition

Both our *ex vivo* and *in vivo* studies were conducted using the same generation of CLE imaging system, making the comparisons reasonable. However, there are some differences in the image acquisition process. First, the average time interval between FNa dosing and imaging is significantly longer in the *ex vivo* study than that in the *in vivo* study (73.2 [55.4] vs. 37.2 [37.8], p = 0.001). In fact, 63.5% of the interpretable images from the *ex vivo* study were acquired 90 minutes or more after FNa dosing. In comparison, 81.0% of the interpretable images from the *in vivo* study were acquired within 60 minutes or less after FNa dosing. This discrepancy may be part of the reason for the substantial difference in image quality. But the intrinsic difference between the two modalities may be better revealed by the disparity of image quality of those acquired 120 minutes or more after FNa dosing from the two studies.

Secondly, for the *ex vivo* study, the CLE probe was used in a fixed position with a probe holder, which provided more stability compared to the hand-held fashion in the *in vivo* study. Furthermore, the *ex vivo* environment allows adjustment of the probe in the best possible position during the scanning process of the resected tissue. This may account for the higher percentage of unusable images from the *in vivo* study, where adjustments to the probe position are made with respect to the surrounding brain tissue and are affected by being hand-held by the surgeon (*in vivo*: 31.1% vs. *ex vivo*: 18.5%). During the *in vivo* scanning process, movement between probe and tissue, imaging depth change, and scanning fiber disturbance introduce artifacts that make the images uninterpretable. Interestingly, in the *in vivo* study, deeply located digital biopsy spots were associated with a higher percentage of interpretable images than superficial ones, which may be attributed to more support of the probe and less range of scanning movement allowed when used on deeper spots (18, 20).

Thirdly, the gain parameter was automatic for the *ex vivo* study and set at 2400 for the *in vivo* study. A gain of 2400 produces clear CLE images in tissues with average fluorescence brightness. Lowering the gain can result in less image oversaturation and noise with intensely fluorescent tissues, thereby raising picture quality. However, with tissues with low fluorescence intensity that produce dark images, increasing gain does improve brightness but lowers overall image quality by enhancing black noise (21).

### Fluorescein sodium pharmacokinetics

The CLE system we used was designed for FNa use only. FNa is a yellow-green fluorescent compound with a major excitation peak in 475-490 nm and a major emission peak in 510-530 nm (33, 34). After intravenous (IV) administration, FNa binds weakly to serum albumin (volume of distribution 0.5 L/kg) and is rapidly distributed throughout the body. FNa undergoes rapid conjugation to fluorescein glucuronide, which also fluoresces (35, 36). Within 10 minutes, the concentration of unbound fluorescein glucuronide exceeds that of unbound fluorescein. The half-lives of FNa and fluorescein glucuronide are 23.5 minutes and 264 minutes, respectively. Hence, fluorescein glucuronide accounts for most of the delayed stage fluorescence. Total plasma fluorescence peaks about 3 minutes after IV injection and then declines slowly. Renal clearance is completed within 24 to 32 hours (37). Fluorescein and fluorescein glucuronide can cross the blood-brain barrier disrupted by certain tumors (27, 38–40), making it an ideal option for CLE imaging of brain tumors.

### CLE image quality and timing of imaging

A previous study reported that *ex vivo* image quality gradually decreased with time after FNa administration, likely due to photobleaching, resulting in darker images with less contrast (19). This analysis, consistent with the pharmacokinetics of FNa, shows a time-dependent decrease in image contrast in the *ex vivo* images. Although not statistically significant, a similar decrease occurred in image brightness with a longer time interval between imaging and the last FNa dose. These findings support the conclusion by Abramov et al. that redosing FNa during the surgery was beneficial for maintaining *ex vivo* image quality and resulted in higher diagnostic accuracy (19).

Correlation analysis between *in vivo* image quality and time from the last FNa dose failed to demonstrate a similar result. In other words, the brightness and contrast of the images acquired *in vivo* did not decrease significantly over time. However, the comparison between *in vivo* images acquired earlier than 120 minutes after FNa dose with those acquired later than 120 minutes showed a 6.3% (112.2 [27.0] to 105.1 [18.9], p < 0.001) decrease in brightness and an 8.2% (44.74 [8.0] to 41.07 [8.6], p < 0.001) decrease in contrast (**Figure 5B**). Since limited conventional biopsy samples were obtained at optical biopsy spots acquired later than 120 minutes after the FNa dose, a comparison of diagnostic performance with respect to CLE imaging timing could not be achieved. In a recent study of *in vivo* CLE imaging with FNa by Höhne et al., the authors reported, though not quantified, that timing of FNa injection did not impact image quality significantly (41).

### FNa redosing

A previous study compared image quality from *ex vivo* CLE imaging before and after FNa redosing, showing that an additional dose of FNa in the later stage of surgery led to a significant improvement in image quality (19). The increase in tissue fluorescence after FNa redosing led to improved brightness and contrast. Here we demonstrated that even with FNa redosing, *ex vivo* images had inferior quality when compared to *in vivo* images (**Figures 2G–L**, **Figure 3**). We postulate that the retained microcirculation of the *in vivo* tissue was essential to maintaining a high enough level of tissue fluorescence that allowed for higher image brightness and contrast. When disconnected from such microcirculation, the *ex vivo* tissue may have undergone considerable fluorescence decay during the short interval between tissue extraction and imaging, making it impossible to produce images with comparable brightness and contrast to the *in vivo* images. However, this theory needs validation with tissue fluorescence measurement and possible animal model simulation.

Redosing of FNa was not done in any of the *in vivo* cases because the surgeons deemed the images of satisfactory brightness and interpretability, so exactly how FNa redosing would affect *in vivo* CLE imaging remains uncertain. Further investigation is required to determine the optimal FNa dosing protocol for *in vivo* CLE imaging in brain tumor surgery.

### Image interpretation and diagnostic performance

**Figure 7:**
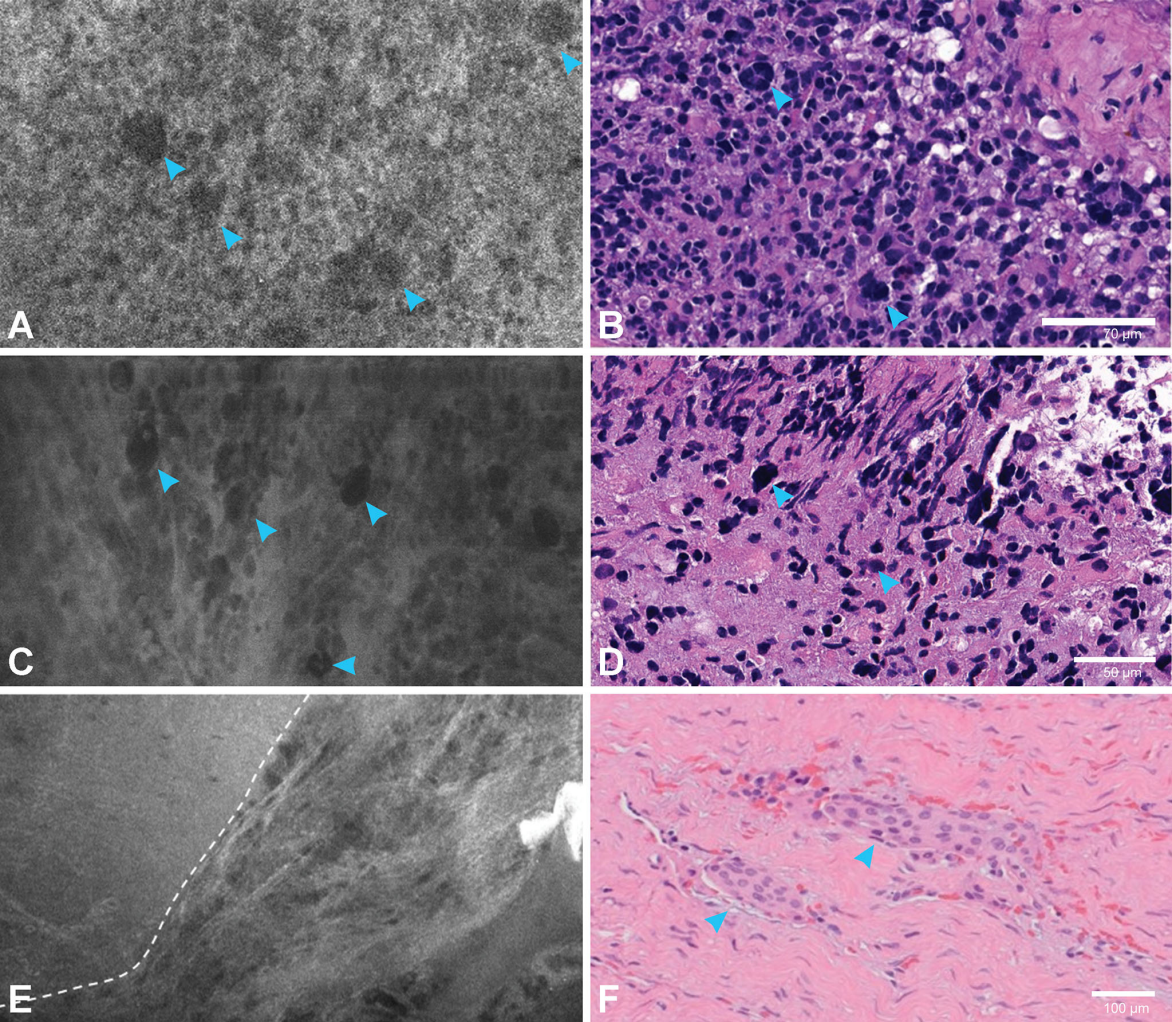
Correlation between confocal laser endomicroscopy (CLE) images and permanent hematoxylin and eosin (H&E)-stained sections. An *in vivo* CLE image from a patient with glioblastoma **(A)** showing hypercellularity and large atypical cells (*arrowheads*) similar to those in the H&E section (*arrowheads*) **(B)**. Another *ex vivo* image from a different patient with glioblastoma **(C)** with hypercellularity and atypical cells (*arrowheads*) also found in the H&E section (*arrowheads*) **(D)**. An *in vivo* image from a patient with meningioma **(E)** demonstrating the clear transition (*dashed line*) of a nest of tumor cells to acellular fibrous dura tissue, with an H&E section **(F)** showing tumor cell nests (*arrowheads*) within normal collagenous dura. *Used with permission from Barrow Neurological Institute, Phoenix, Arizona*.

**Figure 8:**
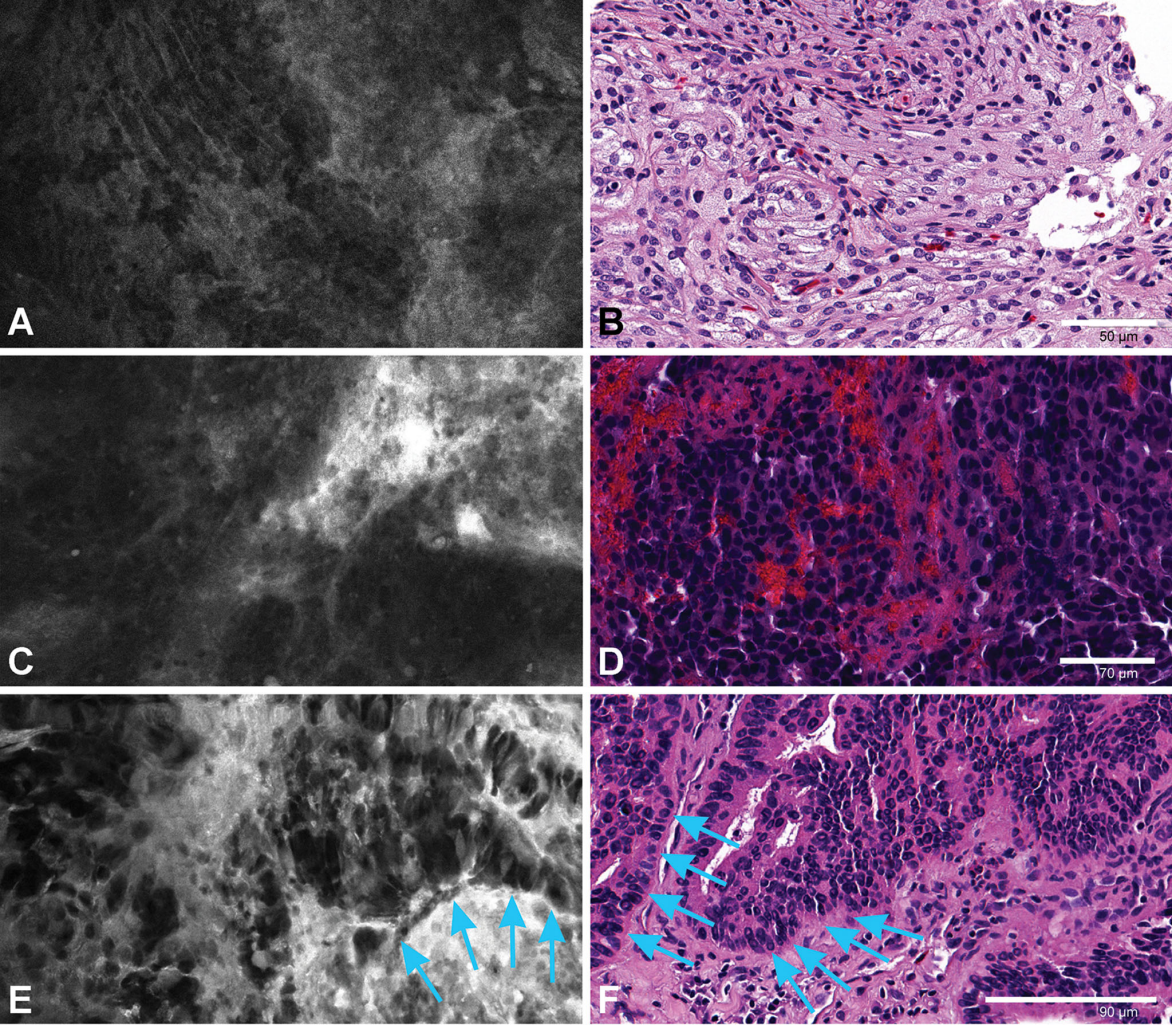
An *ex vivo* meningioma image **(A)** showing refractile fibers and a whirling pattern corresponding to the H&E slide **(B)**. An *in vivo* image from intracranial metastatic breast carcinoma **(C)** with nests of highly dense epithelioid cells. H&E slide of the same patient **(D)** showing tumor cell nests with hemorrhage. An *ex vivo* image **(E)** and H&E section **(F)** from a patient with choroid plexus carcinoma showing tumor cells along the basement membrane (*arrows*). *Used with permission from Barrow Neurological Institute, Phoenix, Arizona*.

For both studies, every single digital biopsy was interpreted by a neuropathologist who was experienced with CLE imaging and blinded to the pathological diagnosis (**Table 2**). Permanent section pathology diagnosis is used as the gold standard (**Figures 7**, **8**). Histopathological features were interpreted on the basis of CLE images, but pathological diagnoses were neither attempted nor made on the basis of CLE images, because we do not have enough data at this early stage to support correlative diagnosis based on the CLE imaging. Lesional CLE digital biopsies were regarded as positive results. Nonlesional and nondiagnostic digital biopsies were regarded as negative results. The *in vivo* study had higher sensitivity and negative predictive value when all pathologies were compared, as well as for the comparison of images of glioma cases separately. This may be because *ex vivo* images are darker and with less contrast, obscuring the cellular and histological structure, making accurate interpretation more difficult, and eventually leading to more false-negative and nondiagnostic results.

One of the challenges of accurate image interpretation is that CLE produces grayscale images instead of the traditional colored H&E sections that physicians see during their education and practice. Previous reports showed that training and experience are necessary to interpret these grayscale images (42, 43). With the application of CLE growing not only in neurosurgery but also in other medical fields such as gastroenterology, ophthalmology, dermatology, etc., more training opportunities are being offered to pathologists (44). Artifacts introduced by blood, air bubbles, or fluid on the imaging surface may mimic tumor cells or other structures that interfere with image interpretation. However, with the help of an innovative telesurgical pathology software platform based on the *in vivo* CLE system that allows pathologists to interpret the scanned tissue in real time and simultaneously interact with a neurosurgeon intraoperatively, such artifact-related disadvantages may be minimized, and the efficiency of diagnosis and surgical decisions enhanced (18, 45, 46).

### CLE imaging for gliomas, meningiomas, and metastases

*in vivo* CLE images have significantly higher brightness and contrast when compared to their *ex vivo* counterparts. This observation is consistent across the three most common intracranial pathology types, namely glioma, meningioma, and intracranial metastatic tumors (**Figure 6**). In fact, the brightness and contrast of *in vivo* images are 65-75% higher than those of *ex vivo* images, except for the contrast of meningioma images. The absolute contrast value of *in vivo* meningioma images (36.7 vs. 43.2 and 49.6) and the difference in contrast between *in vivo* and *ex vivo* images (15.8%) are lower than the other two pathologies. Considering that the contrast is mainly attributable to the variance in the intensity values between the darker cell nuclei and the lighter extracellular matrix background, the smaller difference observed in the contrast of meningioma images could be ascribed to the fact that a considerable percentage of these images represent less cellular dura tissue, i.e., imaging from CLE scanning at the meningioma-dura interface.

CLE imaging of glioma is of particular interest because the marginal regions of glioma are often challenging to discriminate as they transition into surrounding edema and normal brain tissue without the assistance of advanced imaging technology. Previous animal model studies (10, 24) and clinical studies (15) characterized identifiable CLE features of glioma. Combined with image processing techniques like Z-stack and 3D rendering (6), and deep-learning-based image style transfer (47), CLE imaging could improve the accuracy of the tumor mass or resection bed tissue biopsy and histological diagnoses. Moreover, the application of metabolically active fluorophores may further enhance the visualization of cellular structures (48). However, the real value of CLE seems to lie in its potential to help intraoperatively and microscopically delineate the tumor border or tumor resection bed, especially for primary invasive brain tumors with transitional margin regions. Analyses of more CLE images of normal brain, reactive gliotic brain, and marginal tumor infiltration paired with H&E-stained sections of the exact same spot are needed to achieve this level of discernment. This is of paramount importance because *in vivo* CLE imaging could ideally be used multiple times on the resection margin of an intra-axial tumor to assess the presence of residual tumoral tissue.

After the cranium is opened and the brain is exposed, and especially toward the end of tumor resection, brain shift causes significant inaccuracies compared with the neuronavigational system images displayed. Neurosurgeons rely on these images for surgical guidance, but such distortion can cause neuronavigation-image mismatch when determining the exact location for further resection, especially in large or deep glioma resections. CLE imaging seems to have a critical use in exploring the margins or questionable areas in these situations. Its use as a confirmatory surgical tissue biopsy location determinant or optical interrogator paired with rapid next-generation nanopore whole-genome tissue sequencing could provide an efficient yield of tissue that has essential prognostic indications (49).

Pathological features of meningioma, the most common primary intracranial extra-axial tumor, in CLE images are also well characterized in previous studies (9, 16), including refractile fibers, psammoma bodies, intracellular inclusions, and cellular palisading. Dural invasion by nests of cells and cell atypia was observed in non-Grade I meningiomas (16). These features correspond well with H&E slides, making them good diagnostic clues for CLE imaging. Although indicated in one of the *in vivo* meningioma cases, as shown in (**Figure 7E**), where a clear transition of tumor cell nests to acellular fibrous dura tissue is shown, the use of CLE in discriminating meningioma tumor cells invading into the surrounding dura or brain parenchyma needs to be further validated in future studies.

Three cases of intracranial metastatic tumor were included in each study. The features of the CLE images of intracranial metastatic tumor cases include highly dense atypical cells, vacuolated cytoplasm, foci of fibrosis and hemorrhage, and neoplastic blood vessels (9). However, due to the difference in the primary tumors, the CLE images of intracranial metastatic tumor cases are highly heterogeneous.

### Study limitations

Limited by the retrospective nature of this study, certain variables cannot be controlled. The CLE gain setting was different for the two studies. The brightness and contrast measured by Fiji software are based on the gray value of individual pixels. They are close approximations rather than the actual brightness and contrast of the images since, in the images, the cells and other pathognomonic structures are made up of multiple adjacent pixels. To what extent did this difference affect the brightness and contrast values measured by Fiji software cannot be quantified. Nonetheless, this is a reasonable and validated method that reflects gross differences in image quality.

Only images from glioma, meningioma, and intracranial metastatic tumor cases were compared between the two studies. Intraoperative CLE visualization of glioma boundaries provides unique value to assisting tumor resection. Meningioma may warrant this imaging assistance; especially in higher grade meningiomas, CLE may be helpful for discriminating nests of meningioma cells invading the surrounding dura or brain parenchyma, which usually become locations of tumor recurrence. The case numbers for other pathologies are too small to draw meaningful conclusions.

## Conclusions

In our setting, *in vivo* CLE optical biopsy outperforms *ex vivo* CLE by producing higher quality images and less image deterioration, leading to better diagnostic performance. These results support the use of *in vivo* as the modality of choice for intraoperative CLE imaging. Given the similarities in histopathologic features seen on CLE images when compared to classic histopathology, the learning curve for neuropathologists is not steep. The results of this study lay a foundation to initiate larger-scale examinations of dosing, timing, and CLE imaging system parameters correlated to the quality of images, especially for *in vivo* use. CLE imaging technology has the potential to become a key imaging technology, especially in combination with the intraoperative telesurgical pathology software platform, for the neurosurgical operating room.

## Data availability statement

The raw data supporting the conclusions of this article will be made available by the authors, without undue reservation.

## Ethics statement

The studies involving human participants were reviewed and approved by the Institutional Review Board for Human Research at St. Joseph’s Hospital and Medical Center. The participants provided their written informed consent to participate in this study.

## Author contributions

Study planning and coordination, MCP, YX, and IA. Data acquisition, EB, IA, and MTP. Processing and analyzing of data and confocal images, YX, EB, IA, and MCP. Statistical analysis, YX. Drafting the manuscript, YX, IA, GM-J, and MCP. Review of the draft, all authors. Final approval, MCP. All authors contributed to the article and approved the submitted version.

## Funding

This study was supported by funds from the Newsome Chair in Neurosurgery Research held by MCP.

## Acknowledgments

We thank the staff of Neuroscience Publications at Barrow Neurological Institute for assistance with manuscript preparation. Dr. Belykh’s current affiliation is the Department of Neurosurgery, Rutgers New Jersey Medical School, Newark, New Jersey.

## Conflict of interest

The authors declare that the research was conducted in the absence of any commercial or financial relationships that could be construed as a potential conflict of interest.

## Publisher’s note

All claims expressed in this article are solely those of the authors and do not necessarily represent those of their affiliated organizations, or those of the publisher, the editors and the reviewers. Any product that may be evaluated in this article, or claim that may be made by its manufacturer, is not guaranteed or endorsed by the publisher.

## Abbreviations

5-ALA, 5-aminolevulinic acid; CLE, confocal laser endomicroscopy; FDA, Food and Drug Administration (United States); FNa, fluorescein sodium; H&E, hematoxylin and eosin; ICG, indocyanine green; SD, standard deviation.
